# Pain After Transabdominal Preperitoneal (TAPP) or Totally Extraperitoneal (TEP) Technique for Unilateral Inguinal Hernia: A Randomized Controlled Trial

**DOI:** 10.7759/cureus.24582

**Published:** 2022-04-29

**Authors:** Mahaveer S Rodha, Satya P Meena, Krashankant Premi, Naveen Sharma, Ashok Puranik, Ramkaran Chaudhary

**Affiliations:** 1 General Surgery, All India Institute of Medical Sciences, Jodhpur, IND

**Keywords:** tep, tapp, pain, operative time, laparoscopy, inguinal hernia, hospital stay

## Abstract

Introduction

Laparoscopic inguinal hernia repair is the most commonly performed surgery in many hospitals. This study aimed to compare the outcome of the transabdominal preperitoneal (TAPP) and totally extraperitoneal (TEP) techniques in unilateral, uncomplicated inguinal Hernia.

Material and methods

This prospective randomized study was conducted in a tertiary care hospital in North India from November 2018 to March 2020. Sixty-eight male patients of unilateral, uncomplicated inguinal hernia were enrolled for laparoscopic hernia repair. The first group of 34 patients underwent TAPP repair and the second group of 34 patients underwent TEP repair under general anesthesia (GA). Both groups were compared for intraoperative or postoperative complications, analgesic requirements, postoperative pain, length of hospital stay, resumption of routine activity, and patient satisfaction scores. Fisher's exact test or Chi-square test were used for nominal data and the median or interquartile range was used for ordinal data.

Results

The mean operative time for TAPP was more than that for the TEP group (101 vs 76, p<0.001). The TAPP group exhibited significantly less postoperative pain at six hours, 24 hours and seven days than TEP (p<0.001) and an insignificant difference at three months of the follow-up period (p=0.188). Additional analgesics requirement was less in the TAPP group, although the difference was not significant (p=0.099). Seroma formation was found in four patients (11.8%) in the TEP group and two patients (5.9%) in the TAPP group (p= 0.672). Length of postoperative hospital stay (p=0.907), resumption of routine activity (p=0.732), and patient satisfaction scores (p=0.492) during follow-up were similar in both groups and were also insignificant.

Conclusion

The TAPP technique is slightly better than TEP for inguinal hernia in terms of lesser postoperative pain with similar chances of complications and other outcomes.

## Introduction

Laparoscopic hernia repair was first performed by Ger et al. in 1990 [1]. Various techniques are used to do hernioplasty, like laparoscopic transabdominal preperitoneal (TAPP), totally extraperitoneal (TEP), or robotic TAPP. All techniques have the basic principle of placing a synthetic mesh in the preperitoneal space [2-4].

Previous literature has debatable pain outcomes. The TAPP technique resulted in more pain in early postoperative periods as reported by Krishna et al. and Bansal et al. Many other studies have found no difference in pain between TAPP and TEPP procedures [5-8].

The aim of this study was to compare the outcome of TAPP and TEP techniques in unilateral, uncomplicated inguinal hernia.

## Materials and methods

Study design and setting

A prospective randomized controlled trial (RCT) of parallel design, was conducted at the All India Institute of Medical Sciences (AIIMS), a tertiary care teaching hospital in Rajasthan, India. The study was approved by the Institutional Ethical Committee (AIIMS/IEC/2018/619) and Clinical Trials Registry India (CTRI/2018/11/016348).

Study duration and sample size

**Figure 1:**
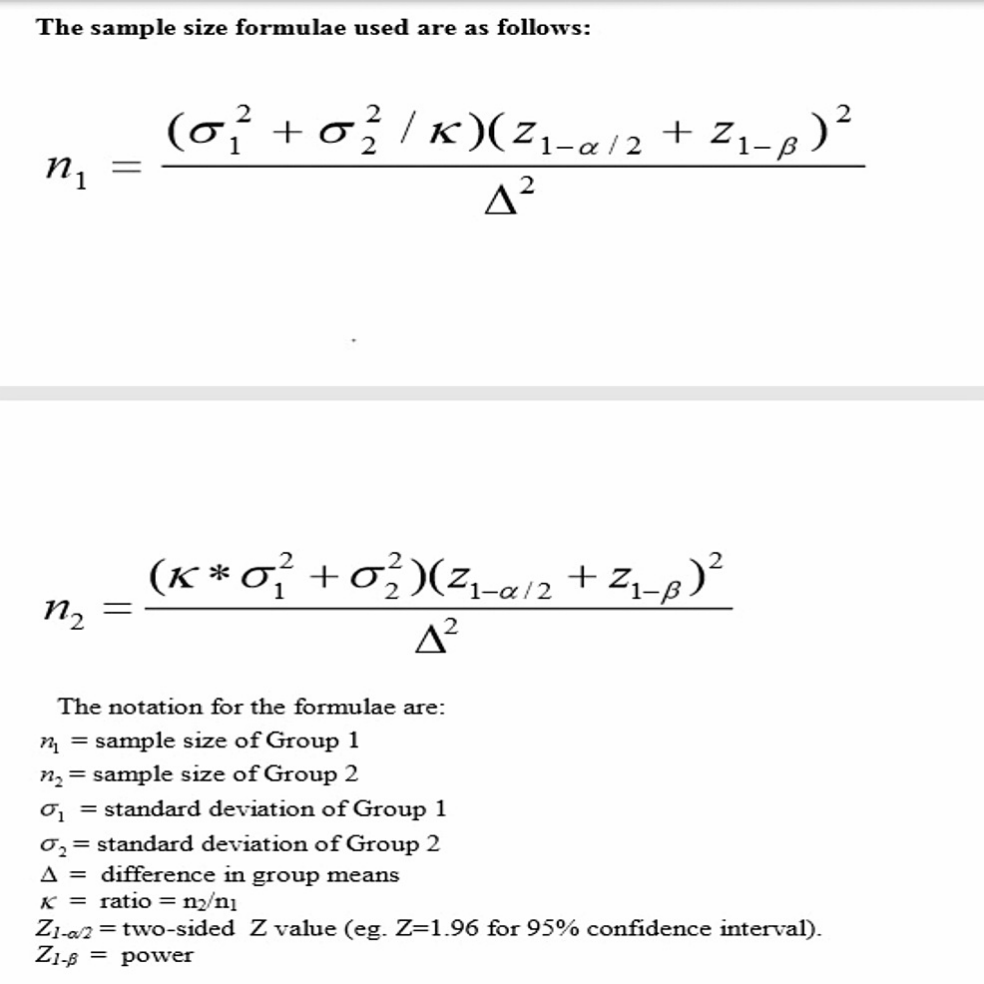
Sample size calculation

The study was started on 23, November 2018, and was completed in 16 months and 17 days. A total of 68 patients were included in this study, who underwent elective laparoscopic inguinal hernia repair. The patients were randomized into two groups. The first group included 34 patients who underwent the TAPP procedure. The second group included 34 patients who underwent the standard TEP technique. The sample size was calculated by considering the mean pain score at three months as 0.96+/- 0.4 in the TEP group and 1.28 +/- 0.45 in the TAPP group. We calculated the sample size according to the study conducted by Krishna et al., and 34 patients were included in each group at 80% power, 95% confidence interval and 20% contingency for attrition (Figure 1 ) [5-8].

Study participants

This study included male patients aged over 18 years with a reducible unilateral inguinal hernia undergoing elective laparoscopic hernia repair (TEP or TAPP). This study excluded all patients who were not willing to participate in the study, bilateral hernias, complicated or recurrent inguinal hernia, coagulopathy, previous history of lower abdominal surgery and high-risk patients for general anesthesia (GA) due to comorbidities like coronary artery disease (CAD), chronic obstructive pulmonary disease (COPD), chronic kidney disease (CKD), chronic liver disease (CLD).

**Figure 2:**
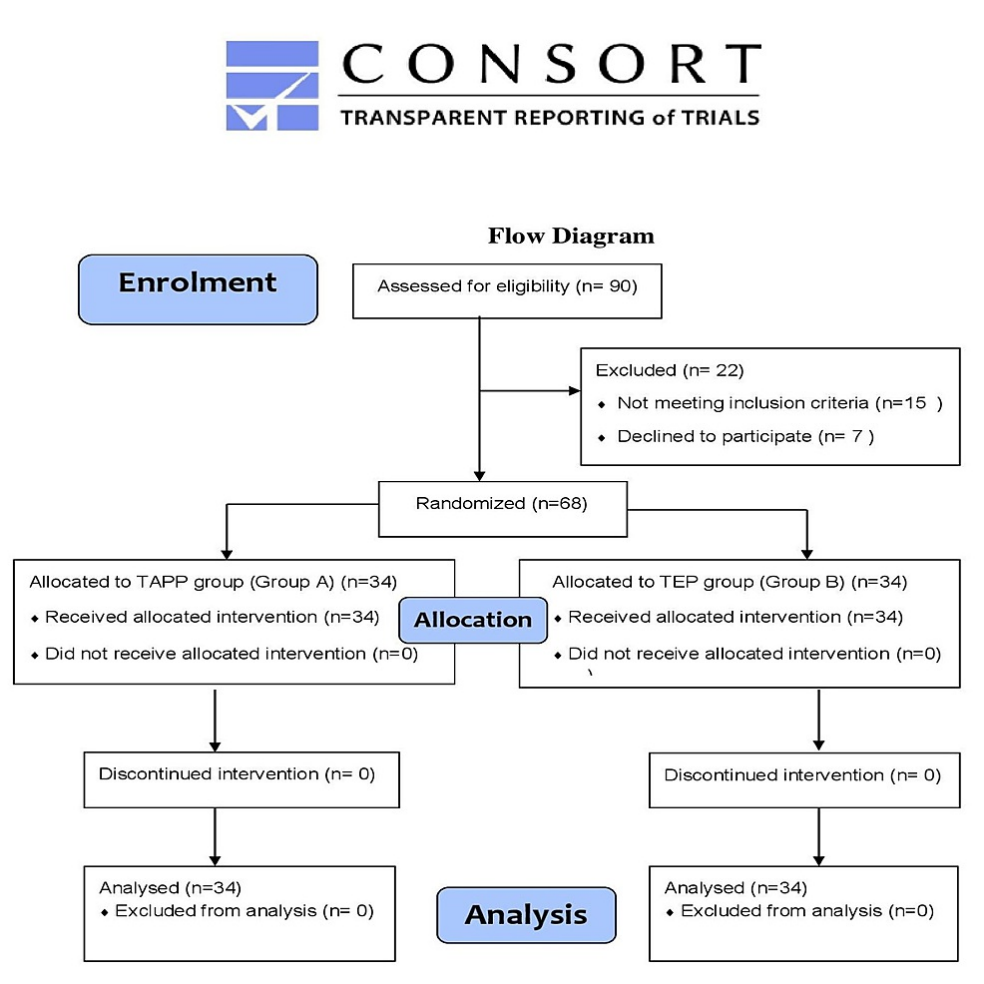
The CONSORT flow diagram CONSORT: Consolidated Standards of Reporting Trials

The Consolidated Standards of Reporting Trials (CONSORT) flow diagram depicting the progress of the parallel RCT is shown in Figure 2.

Objectives

This study's primary objective was to compare postoperative pain between the TAPP group and the TEP group. The secondary objective was to compare operative duration, intraoperative complications, postoperative seroma, hematoma formation or infection rate, hospital stay and resumption of routine work.

Randomization

Each patient was randomly allocated to the TAPP group or the TEP group by sequentially numbered, opaque, sealed envelope (SNOSE) technique on the day of surgery. An independent statistician, who was not involved in patient care, generated the randomization sequence via a computer-generated random number. The randomization code was contained in opaque sealed envelopes. A non-acad junior resident, who opened the envelope, was not actively involved in outcome measurements. Before randomization and during consent, all patients received an explanation of the objectives of the study, techniques and complications associated with both procedures. The patient was unaware of the assigned group of treatment. The allocation ratio was 1:1 for this RCT study.

Surgical procedure

In the preoperative area, a single prophylactic dose of injection ceftriaxone 1 gm was intravenously administered after the skin test. All patients had a urinary catheter. All procedures were performed under GA with the patients in supine and Trendelenburg position as per the conventional three ports technique. The patients were randomized into two groups:

TAPP Technique

Pneumo-peritoneum was created through the supraumbilical port using a Verres needle after induction of GA. After obtaining intra-abdominal pressure of 14 mm of Hg, one 10 mm camera port was placed in the supraumbilical region and the remaining two 5 mm ports were kept in the bilateral mid-clavicular line at the level of the umbilicus. After inspection of the abdomen, a 5 cm peritoneal incision was made from the cranial to inguinal defect. The Cooper’s ligament was identified medially during pre-peritoneal dissection. The medial limit of dissection was Cooper’s ligament of the opposite side. Cord structures were identified and the hernia sac was separated from cord structures. The anterior superior iliac spine (ASIS) of the ipsilateral side was the lateral limit of dissection. The lower limit of dissection was where vas deferens turn medially. After proper dissection, a 15x12 cm polypropylene mesh was placed in the pre-peritoneal space. The peritoneal flap was sutured with absorbable suture followed by port site closure with non-absorbable nylon suture after closing the supraumbilical fascial defect with polyglactin suture.

TEP Technique

A 10 mm port was placed just below the umbilicus for the 10 mm 30 ^0^ telescope after the induction of GA. A preperitoneal space was created with the help of telescopic blunt dissection until the pubic symphysis was seen in the midline. Further dissection proceeded with another two 5 mm working ports, one just above the pubic symphysis and the other in the midline between the umbilical port and pubic symphysis. The lateral limit of preperitoneal flap dissection corresponded to the anterior superior iliac spine. The peritoneum was teased down as low as possible with careful dissection to expose the psoas major muscle, the nerves, deep ring and triangle of doom. After the reduction of the hernial sac, a 15x12 cm polypropylene mesh was unrolled in the preperitoneal space to adequately cover the possible hernial sites and was not fixed with any suture or clips. One or two interrupted sutures with polyglactin were applied for umbilical fascial closure after releasing the pneumoperitoneum.

Post-operative period

The urinary catheter was removed immediately after the completion of the procedure. Post-operatively, an injection of paracetamol 1 gram was infused intravenously every eight hours on the day of surgery in all patients of both the groups as per the standard protocol of our department. The additional analgesic requirement was fulfilled by injection of diclofenac 75 mg by the intravenous route, as needed if the visual analog scale (VAS) score was more than three. An assessment of pain was made using the VAS score in the postoperative and follow-up periods. 

Statistical analysis

We collected data in the paper-based case record form before, during and after operations and transferred this data to Microsoft Office Excel. A statistician, who was not involved in patient care, performed the statistical analysis. Statistical analysis was done by using the Statistical Package for the Social Sciences (SPSS) software version 23.0 (IBM Corp, Armonk, NY, USA). Nominal data like complications present or absent were described using frequency and percentages and compared using the Chi-square test or Fisher's exact test. Ordinal data like VAS scores were described using median and interquartile range (IQR) and compared using the Mann-Whitney U test. Continuous data like operative time were described using mean ± SD and compared using the unpaired t-test. A P-value of <0.05 was considered statistically significant.

## Results

Demographic profile

Sixty-eight male patients were randomized with the diagnosis of reducible unilateral primary inguinal hernia in TEP and TAPP groups. There were two smokers in each group. In the TAPP group, two patients had an addiction to alcohol and opium. Three patients in the TEP group and one patient in the TAPP group were diabetic. Four patients in the TEP group and five patients in the TAPP group were hypertensive. One patient in the TEP group had COPD. The demographic profiles were comparable between the two groups and had no significant impact on outcomes in either group (Table 1).

**Table 1:** Comparison of demographic profile between the TEP and TAPP TEP: Totally extraperitoneal repair, TAPP: Transabdominal preperitoneal repair, IQR: Interquartile range

Demographic profile		TAPP (n-34)	TEP (n-34)
Age	Median (IQR)	49.0 (40.0, 62.0)	51.0 (33.0, 60.0)
Type of hernia	Direct	15 (44.12%)	6 (17.65%)
	Indirect	19 (55.88%)	28 (82.35%)
Laterality	Right	23 (67.65%)	26 (76.47%)
	Left	11 (32.35%)	8 (23.53%)
Extent	Incomplete	26 (76.47%)	30 (88.24%)
	Complete	8 (23.53%)	4 (11.76%)

Operative time, length of hospital stay, and resumption of routine activity

The median operative time in the TAPP group was 101.0 minutes while in the TEP group it was 76.5 minutes. The operative time in the TAPP group was 24.5 minutes longer as compared to the TEP group (p < 0.001). Operative time is the time taken from skin incision to skin closure. The length of hospital stay was not statistically significantly different in the two groups (p value=0.907). The patients in both groups resumed routine activities after 30 days of operation (p-value 0.732) and were also not significant (Table 2).

**Table 2:** Comparison of the intraoperative time, length of hospital stay, and resumption of routine activity TEP: Totally extraperitoneal repair, TAPP: Transabdominal preperitoneal repair, IQR: Interquartile range

Outcome	TAPP (n=34)	TEP (n=34)	
	Median (IQR)	Median(IQR)	P-value
Operative time	101.1(88, 115)	76.5 (68, 85)	<0.001
Length of hospital stay (Hours)	29 (27, 49)	29 (27, 48)	0.907
Resumption of routine activity (Days)	30 (30, 40)	30 (25, 60)	0.732

Postoperative pain

It has been observed that additional analgesia (diclofenac 75 mg) was required in six patients of TAPP and twelve patients of the TEP group within six hours of surgery due to excessive postoperative pain (p-value=0.099). Analgesics such as morphine were not administered to any of the patients during surgery. The median VAS score on postoperative one hour, six hours, 24 hours and the seventh day was compared in both groups. The median VAS score at one hour was comparable and insignificant (p-value 0.429). The median VAS score at six hours, 24 hours and at the seventh day was more in TEP groups as compared to the TAPP group which was statistically significant (p-value <0.001). After three months of follow-up, the difference in the VAS score was comparable and insignificant between the two groups (p=0.188). None of the patients in either group had chronic groin pain (Table 3).

**Table 3:** Comparison of postoperative pain and follow-up pain (VAS score) VAS: Visual analog scale, TEP: Totally extraperitoneal repair, TAPP: Transabdominal preperitoneal repair, IQR: Interquartile range

VAS	TAPP	TEP	P-value
	Median (IQR)	Median (IQR)	
VAS @ 1 hr	6 (5,6)	6 (5,6)	0.429
VAS @ 6 hrs	3 (3,4)	4 (4,5)	<0.001
VAS @ 24 hrs	2 (1,2)	3 (2,3)	<0.001
VAS score @ 7 days	0 (0, 0.25)	1 (0,2)	<0.001
VAS @ 3 months	0 (0,0)	0 (0,0)	0.188

Complications

No intraoperative complications occurred in the TAPP group, however, one patient had a urinary bladder injury in the TEP group which was managed conservatively. In the TEP group, one patient had developed a left-sided scrotal hematoma, which was diagnosed by ultrasonography on postoperative day seven and was managed conservatively. Four patients in the TEP group and two patients in the TAPP group developed seroma, however, no statistically significant difference was found in both groups. The seroma was assessed clinically. One patient in the TAPP group had complaints of recurrence of right-sided inguinal hernia with subacute intestinal obstruction after two months of operation (Table 4 ).

**Table 4:** Postoperative complications of direct and indirect type hernia in TAPP and TEP groups VAS: Visual analog scale, TEP: Totally extraperitoneal repair, TAPP: Transabdominal preperitoneal repair

Postoperative complications	TAPP (n=34)		TEP (n=34)		P-value
	Direct (15)	Indirect (19)	Direct (6)	Indirect (28)	
Pain at 6 hours (VAS >3)	4	10	2	19	<0.001
Urinary bladder injury	0	0	1	0	0.50
Scrotal hematoma	0	0	0	1	0.50
Seroma	1	1	1	3	0.43
Intestinal obstruction	1	0	0	0	0.50
Recurrence	0	1	0	0	0.50

Patient satisfaction score

**Figure 3:**
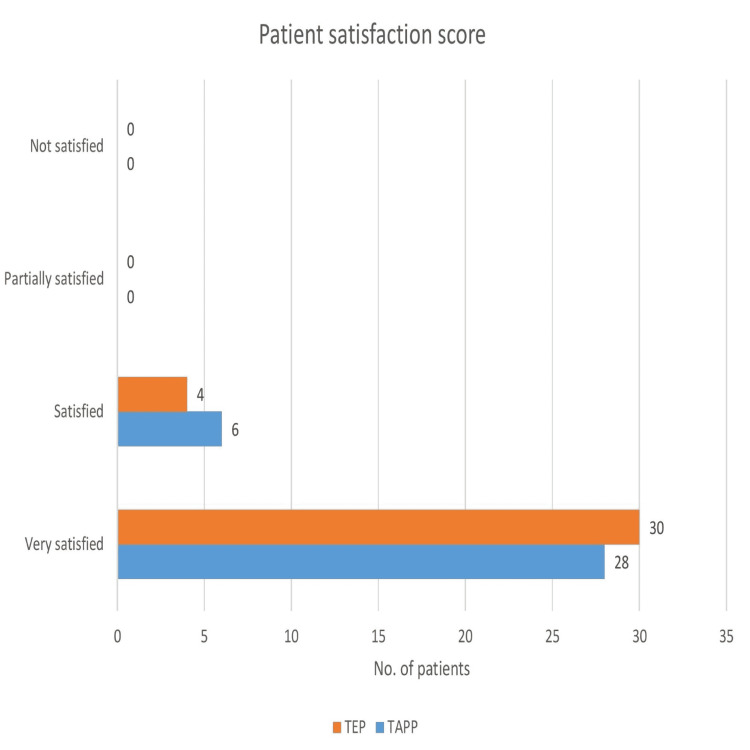
Patient satisfaction score TAPP: Transabdominal preperitoneal repair,  TEP: Totally extraperitoneal repair

Patient satisfaction score (Figure 3) was calculated with the help of a verbal rating scale (VRS) after surgery, where 0=not satisfied, 1=partially satisfied, 2=satisfied and 3=very satisfied. In both groups, not a single patient was either partially satisfied or unsatisfied. The satisfaction score was not statistically significant between the two groups (p=0.492, Chi-square = 0.47).

## Discussion

The present study is a hospital-based RCT to compare outcomes in both techniques of laparoscopic hernia repair. In this study, a statistically significant difference was observed in operative time between the two groups. The reason for the longer operative time for TAPP in our study could be due to the suturing of the peritoneum to cover the mesh. This result was consistent with those of the previous study [9]. However, a study by Gong et al. which included uncomplicated unilateral inguinal hernia, and Sharma et al. which included uncomplicated bilateral inguinal hernia, found that operative time in TEP was more compared to TAPP [10,11]. However, in both of these studies, the difference was statistically insignificant. Reasons for an increased intraoperative time in TEP given by them were limited working place and difficulty in appreciation of anatomical landmarks.

Pain is one of the commonest and most troublesome complaints in the postoperative period in hernia surgery. In our study, we observed significantly more pain in the TEP group compared to the TAPP group. The median VAS score was statistically higher in the TEP group till the seventh postoperative day followed by no significant difference observed in the next three months. The additional analgesic requirement was higher in the TEP group as compared to the TAPP group. These results were consistent with other studies [11]. In our study, the reason for more pain in the TEP group could be extensive dissection from the umbilicus up to the pubic symphysis. Another reason for more pain in the TEP group could be due to the greater proportion of indirect inguinal hernias operated in this group as compared to the TAPP group. According to Sharma et al., indirect inguinal hernia had higher postoperative pain compared to direct inguinal hernia [11]. The study by Varcus et al. found no significant difference in postoperative pain between TAPP and TEP groups [12]. However, one study has shown that the pain in the TAPP group was higher compared to the TEP group [13].

The duration of hospital stay adds a financial burden to the patient and the hospital. Laparoscopic hernia repair has reduced the postoperative hospital stay as compared to open hernia repair as it is a minimally invasive procedure. In our study, the median length of hospital stay was comparable in both groups (p-value=0.907). The reason for no significant difference in hospital stay in our study could be due to two reasons. First, most of our patients were from far away and remote places so they were not discharged at the earliest in both the groups. Our results were consistent with three other studies [11,14-16]. Two studies have reported a longer duration of hospital stay in the TAPP group compared to the TEP group. The reason for longer hospital stays in the TAPP group in the study by Kockerling et al. was due to surgery for larger defects and a greater number of complete hernia cases in the TAPP group as compared to the TEP group [17,18]. The higher incidence of postoperative complications also leads to a longer duration of hospital stay. In a study by Sudarshan et al., the TAPP group had a longer duration of hospital stay compared to the TEP group though the reason behind the longer hospital stay in the TAPP group was not explained [19]. In the meta-analysis by Bracale et al., there was a significantly longer postoperative hospital stay in the TAPP group [20].

In this study, one patient had intraoperative complications in the TEP group and one patient had postoperative complications in each group. However, the difference was statistically insignificant [14,16,19,20]. Although seroma formation was found twice in the TEP group, it was diagnosed clinically and managed conservatively. Seroma formation occurs more commonly in complete hernia sacs that have undergone extensive dissection and are more common in TEP than in TAPP. No significant seroma difference was found in Sudarshan et al.'s study [16]. A similar finding was observed in the study by Lau et al. [21]. There was no conversion from TEP to TAPP or open hernia repair in either group. However, a few studies have found more conversion in TEP groups [15,18,22].

The resumption of routine activity is an important measure to assess the success of any surgical intervention. The resumption of routine activity would indirectly measure the social and economic impact of the procedure on society. The median time of resumption of routine activity was similar in both groups in our study. The resumption of routine activity was statistically not significant in the two groups (p-value=0.732). Three studies have shown similar outcomes [11,14-16,19,22,23]. All the patients did well at seven days and the three-month follow-up. They were evaluated by clinical examination in their follow-up visits. The patient satisfaction score was insignificant between the two groups. Most of the patients were very satisfied in both the laparoscopic procedure groups and a few were only satisfied but no one came under the category of partially satisfied or not satisfied [23].

The limitation of this study was the shorter duration of follow-up to deduce late postoperative pain and recurrence. Although failed primary repair may be a rare cause for early presentation of recurrence. Further studies with at least a one-year duration of follow-up may provide more evidence regarding the superiority of one technique over the other. The meta-analysis may have high reliability for the outcome of both techniques.

## Conclusions

The TAPP technique has a longer intraoperative time but less pain in the early postoperative period. However, there is no significant difference in pain after one week of surgery in both groups. Other factors like length of hospital stay, postoperative seroma or hematoma formation, additional analgesic requirement, resumption to normal daily activity and patient satisfaction scores are similar in TAPP and TEP groups. The inguinal hernia patient may consider the TAPP procedure as a laparoscopic choice for smooth recovery in the early postoperative period. 

## References

[1] Ger R, Monroe K, Duvivier R, Mishrick A (1990). Management of indirect inguinal hernias by laparoscopic closure of the neck of the sac. Am J Surg.

[2] Neumayer L, Giobbie-Hurder A, Jonasson O (2004). Open mesh versus laparoscopic mesh repair of inguinal hernia. N Engl J Med.

[3] Jacob BP, Ramshaw B (2013). The SAGES manual of hernia repair. Springer New York Heidelberg Dordrecht London..

[4] Miller HJ (2018). Inguinal hernia: mastering the anatomy. Surg Clin North Am.

[5] Krishna A, Misra MC, Bansal VK, Kumar S, Rajeshwari S, Chabra A (2012). Laparoscopic inguinal hernia repair: transabdominal preperitoneal (TAPP) versus totally extraperitoneal (TEP) approach: a prospective randomized controlled trial. Surg Endosc.

[6] (2022). OpenEpi Menu. http://openepi.com/Menu/OE_Menu.htm.

[7] Zhong B (2009). How to calculate sample size in randomized controlled trial?. J Thorac Dis.

[8] Walters SJ, Jacques RM, Dos Anjos Henriques-Cadby IB, Candlish J, Totton N, Xian MT (2019). Sample size estimation for randomised controlled trials with repeated assessment of patient-reported outcomes: what correlation between baseline and follow-up outcomes should we assume?. Trials.

[9] Bansal VK, Misra MC, Babu D (2013). A prospective, randomized comparison of long-term outcomes: chronic groin pain and quality of life following totally extraperitoneal (TEP) and transabdominal preperitoneal (TAPP) laparoscopic inguinal hernia repair. Surg Endosc.

[10] Gong K, Zhang N, Lu Y (2011). Comparison of the open tension-free mesh-plug, transabdominal preperitoneal (TAPP), and totally extraperitoneal (TEP) laparoscopic techniques for primary unilateral inguinal hernia repair: a prospective randomized controlled trial. Surg Endosc.

[11] Sharma D, Yadav K, Hazrah P, Borgharia S, Lal R, Thomas S (2015). Prospective randomized trial comparing laparoscopic transabdominal preperitoneal (TAPP) and laparoscopic totally extra peritoneal (TEP) approach for bilateral inguinal hernias. Int J Surg.

[12] Vărcuş F, Duţă C, Dobrescu A, Lazăr F, Papurica M, Tarta C (2016). Laparoscopic Repair of Inguinal Hernia TEP versus TAPP. Chirurgia (Bucur).

[13] Bansal VK, Krishna A, Manek P (2017). A prospective randomized comparison of testicular functions, sexual functions and quality of life following laparoscopic totally extra-peritoneal (TEP) and trans-abdominal pre-peritoneal (TAPP) inguinal hernia repairs. Surg Endosc.

[14] Aiolfi A, Cavalli M, Del Ferraro S (2021). Total extraperitoneal (TEP) versus laparoscopic transabdominal preperitoneal (TAPP) hernioplasty: systematic review and trial sequential analysis of randomized controlled trials. Hernia.

[15] Wake BL, McCormack K, Fraser C, Vale L, Perez J, Grant AM (2005). Transabdominal pre-peritoneal (TAPP) vs totally extraperitoneal (TEP) laparoscopic techniques for inguinal hernia repair. Cochrane Database Syst Rev.

[16] Wei FX, Zhang YC, Han W, Zhang YL, Shao Y, Ni R (2015). Transabdominal preperitoneal (TAPP) versus totally extraperitoneal (TEP) for laparoscopic hernia repair: a meta-analysis. Surg Laparosc Endosc Percutan Tech.

[17] Köckerling F, Koch A, Adolf D, Keller T, Lorenz R, Fortelny RH, Schug-Pass C (2018). Has Shouldice repair in a selected group of patients with inguinal hernia comparable results to Lichtenstein, TEP and TAPP techniques?. World J Surg.

[18] Gass M, Banz VM, Rosella L, Adamina M, Candinas D, Güller U (2012). TAPP or TEP? Population-based analysis of prospective data on 4,552 patients undergoing endoscopic inguinal hernia repair. World J Surg.

[19] Sudarshan PB, Sundaravadanan BS, Prabu Shankar S (2017). A comparative study of totally extraperitoneal versus transabdominal preperitoneal repair of inguinal hernias. Int Surg J.

[20] Bracale U, Melillo P, Pignata G, Di Salvo E, Rovani M, Merola G, Pecchia L (2012). Which is the best laparoscopic approach for inguinal hernia repair: TEP or TAPP? A systematic review of the literature with a network meta-analysis. Surg Endosc.

[21] Lau H, Lee F (2003). Seroma following endoscopic extraperitoneal inguinal hernioplasty. Surg Endosc.

[22] Felix EL, Michas CA, Gonzalez MH Jr (1995). Laparoscopic hernioplasty. TAPP vs TEP. Surg Endosc.

[23] Schrenk P, Woisetschläger R, Rieger R, Wayand W (1996). Prospective randomized trial comparing postoperative pain and return to physical activity after transabdominal preperitoneal, total preperitoneal or Shouldice technique for inguinal hernia repair. Br J Surg.

